# Supplementary material for: Proteolytic Cleavage of Bioactive Peptides and Protease-Activated Receptors in Acute and Post-Colitis
Source: Int J Mol Sci. 2021 Oct 2;22(19):10711. doi: 10.3390/ijms221910711 (PMC8509398; doi:10.3390/ijms221910711)
Supplement: Supplementary file 1 [file ijms-22-10711-s001.zip › ijms-1391673-supplementary.pdf]

Supplementary materials

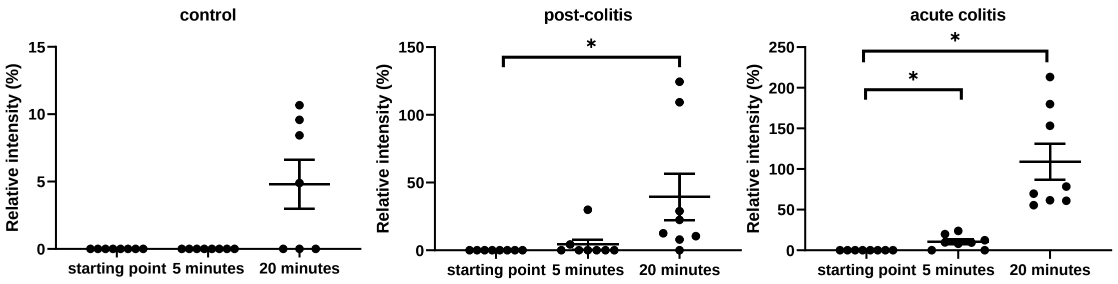

Figure S 1: Bradykinin was processed to BK1-7 by colonic samples from acute and post-colitis rats. The relative intensities of bradykinin(BK)1-7 at the starting point and after 5 and 20 min of incubation with colon samples from control, post-colitis and acute colitis animals are shown here. The increase of BK1-7 is significant after 20 min of incubation in post-colitis samples ($p = 0.02$) and after 5 min of incubation in acute colitis samples ($p = 0.03$). Statistics: Friedman test followed by Wilcoxon signed-rank test with Bonferroni correction. The mean and SEM are shown. n = 8 animals per group *$p<0.05$

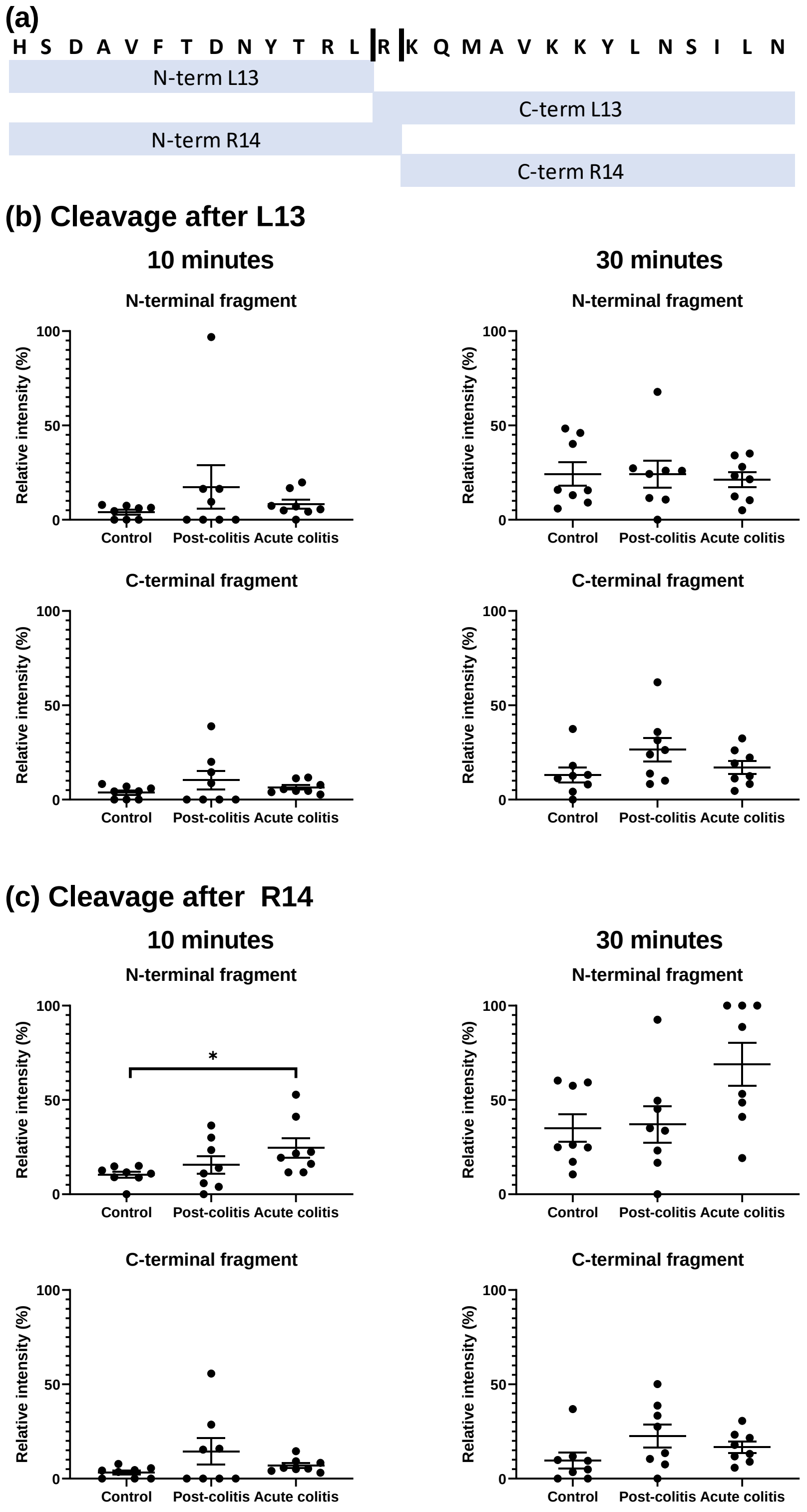

Figure S 2: (a) Cleavage sites in VIP after incubation with colonic samples from control, acute colitis and post-colitis rats. (b) Relative intensities of the N and C-terminal fragment after cleavage at leucine in position 13 after 10 and 30 min of incubation with the colonic samples. (c) Relative intensities of the N and C-terminal fragment after cleavage at arginine in position 14 after 10 and 30 min of incubation with the colonic samples. Statistics: Kruskal-Wallis test with post-hoc Dunnett-Bonferroni. The mean and SEM are shown. n = 8 animals per group *$p<0.05$

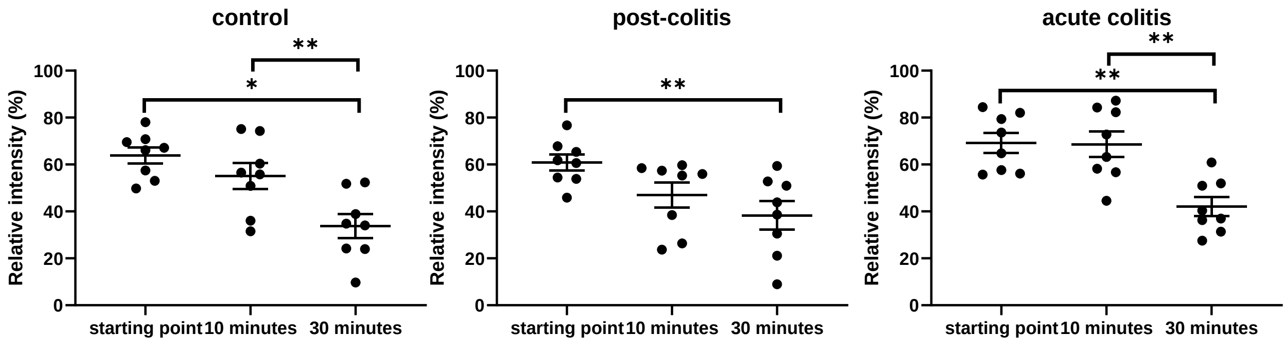

Figure S 3: Relative intensities of the full-length substance P at the starting point and after 10 and 30 min of incubation with colonic samples from control, acute colitis or post-colitis rats. The decrease of the full-length peptide is significant in control, acute and post-colitis samples after 30 min of incubation. Statistics: Friedman test followed by Wilcoxon signed-rank test with Bonferroni correction. The mean and SEM are shown. n = 8 animals per group *p<0.05 **p<0.01

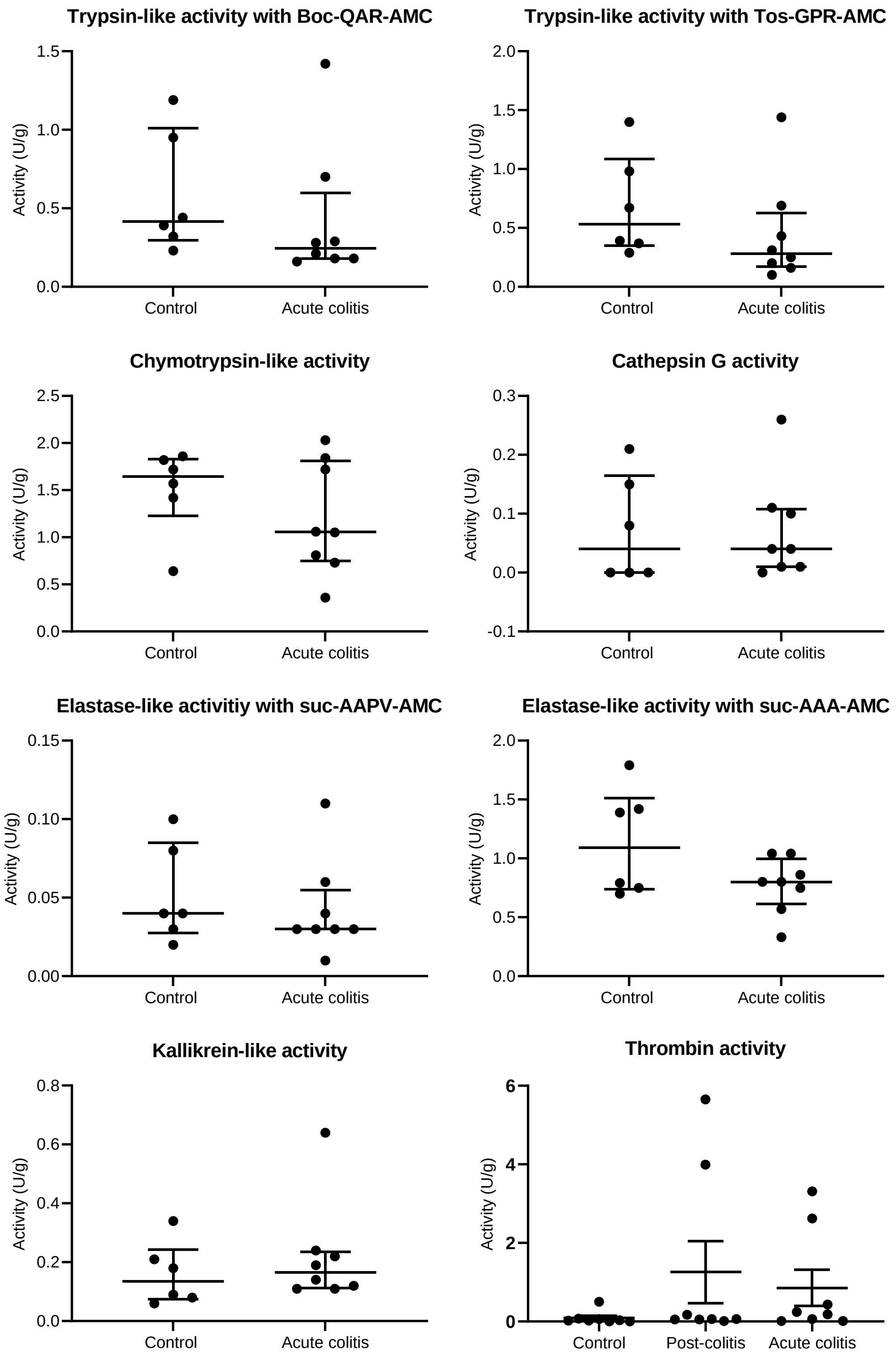

Figure S 4: Proteolytic activities in colon samples from a TNBS-induced colitis model in the acute or post-inflammatory phase measured with fluorogenic substrates. Cathepsin G and chymotrypsin-like activity were measured with suc-AAPF-AMC, elastase-like activity was measured with suc-AAPV-AMC and suc-AAA-AMC, trypsin-like activity was measured with Boc-QAR-AMC and Tos-GPR-AMC, kallikrein activity was measured with H-PFR-AMC and thrombin activity was measured with Tos-GPR-AMC and dabigatran. Statistics: Mann-Whitney U test (with Bonferroni correction for thrombin activity measurement). The mean and SEM are shown. n = 8 animals per group.

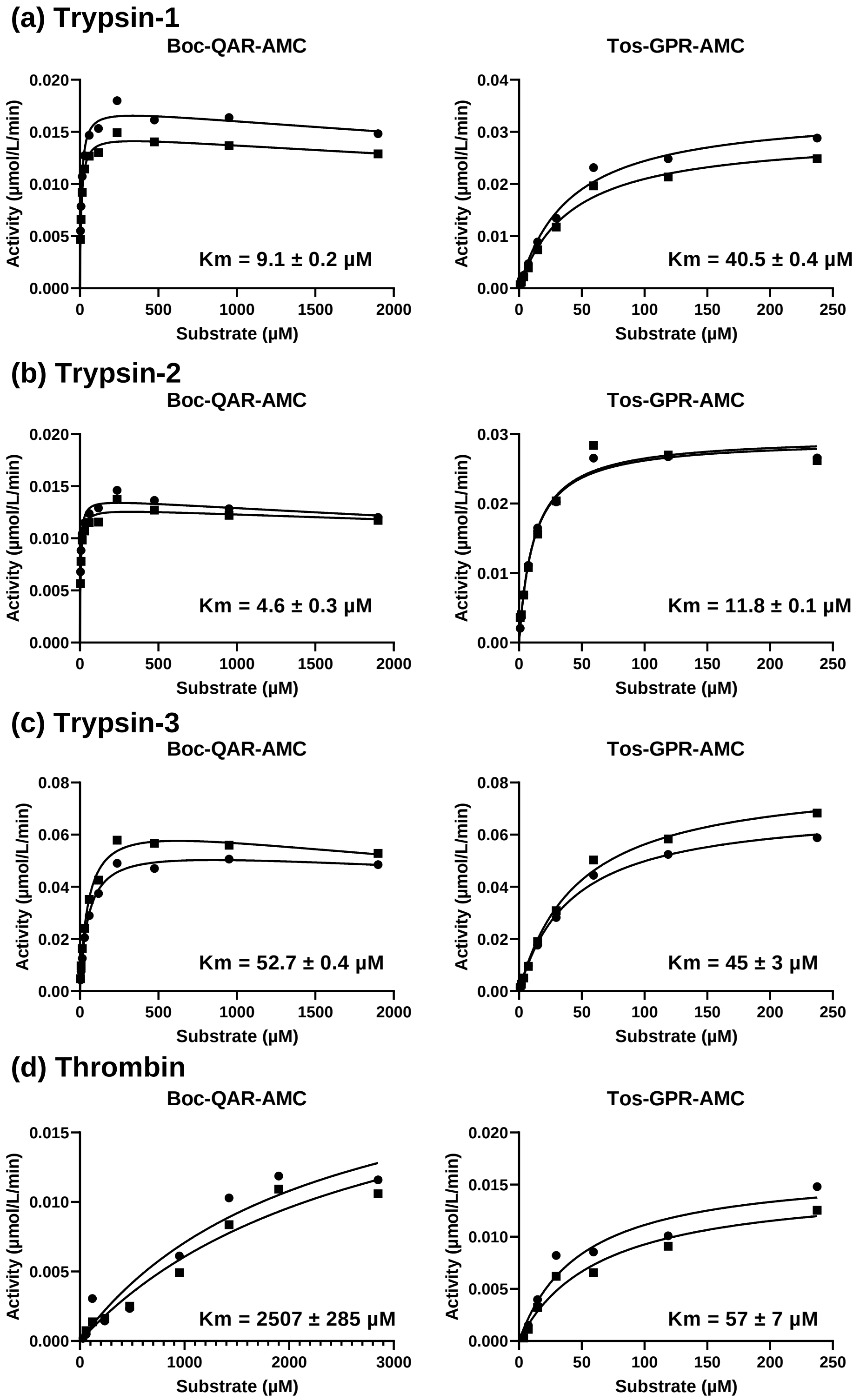

Figure S 5: Michaelis-Menten constant (Km) for (a) trypsin-1, (b) trypsin-2, (c) trypsin-3 and (d) thrombin with Boc-QAR-AMC and Tos-GPR-AMC. The Km was determined with Boc-QAR-AMC and Tos-GPR-AMC by adding trypsin-1 (0.015 nM), trypsin-2 (0.015 nM), trypsin-3 (0.015 nM) or thrombin (0.19 nM) to a range of substrate concentrations at 37°C. The fluorescence was measured kinetically for 20 min at 37°C on a Tecan F200 multi-plate reader. The results in RFU/min were recalculated by dividing them by the slope of a calibration curve with AMC (0.78 – 10 µM) to obtain results in µmol/L/min. These activities were plotted against the substrate concentrations and GraphPad Prism 9.2.0. was used to fit a Michaelis-Menten curve to the data and calculate Km. The experiment was repeated twice.
